# Molecular and Microbial Signatures Predictive of Prebiotic Action of Neoagarotetraose in a Dextran Sulfate Sodium-Induced Murine Colitis Model

**DOI:** 10.3390/microorganisms8070995

**Published:** 2020-07-03

**Authors:** Fang Liu, Jianan Liu, Thomas T.Y. Wang, Zhen Liu, Changhu Xue, Xiangzhao Mao, Qingjuan Tang, Robert W. Li

**Affiliations:** 1 College of Food Science and Engineering, Ocean University of China, Qingdao 266400, China; liufang910205@163.com (F.L.); liuzhenyq@ouc.edu.cn (Z.L.); xuech@ouc.edu.cn (C.X.); 2 Animal Genomics and Improvement Laboratory, Agricultural Research Service, United States Department of Agriculture, Beltsville, MD 20705, USA; liuansc@gmail.com (J.L.); robert.li@usda.gov (R.W.L.); 3 Diet, Genomics and Immunology Laboratory, Beltsville Human Nutrition Center, United States Department of Agriculture, Beltsville, MD 20705, USA; tom.wang@usda.gov

**Keywords:** neoagarotetraose, colitis, gut microbiota, metabolites

## Abstract

Neoagarotetraose (NT), a hydrolytic product of agar by β-agarase, is known to possess bioactive properties. However, the mechanisms via which NT alleviates intestinal inflammation remain unknown. In this study, a dextran sulfate sodium (DSS)-induced murine model was developed to evaluate the effect of NT on gut microbiome and microbial metabolism using 16S rRNA gene sequencing and untargeted metabolomics. Our data demonstrate that NT ingestion improved gut integrity and inflammation scores. NT reversed the abundance of Proteobacteria from an elevated level induced by DSS and significantly increased the abundance of Verrucomicrobia. Further, NT significantly increased the abundance of *Akkermansia* and *Lactobacillus* and concomitantly decreased that of *Sutterella*, which were among the important features identified by random forests analysis contributing to classification accuracy for NT supplementation. A microbial signature consisting of *Adlercreutzia* (denominator) and *Turicibacter* (numerator) predicted the NT supplementation status. Moreover, NT significantly modulated multiple gut metabolites, particularly those related to histidine, polyamine and tocopherol metabolism. Together, our findings provided novel insights into the mechanisms by which NT modulated the gut microbiome and metabolome and should facilitate the development of NT as a potent prebiotic for colitis management.

## 1. Introduction

Inflammatory bowel disease (IBD), a chronic disease partially resulting from altered pre-resolving pathways of intestinal inflammation, is one of the leading global health concerns [1]. Currently, safe and efficacious yet affordable therapeutic options for IBD are limited due to the complex and multifactorial nature of the disease [2]. Decreased alpha diversity, disordered microbial composition and the translocation of the gut microbiome are frequently observed in IBD patients [3,4]. An inappropriate inflammatory response to enteric microbiota is considered one of the key factors driving IBD pathogenesis [5]. Moreover, microbiota-derived metabolites not only provide host energy supplies, but also play a crucial role in immune regulation [6]. For example, metabolites related to tryptophan metabolism, short chain fatty acids (SCFA), bile acids and polyamines are important in IBD pathogenesis [7,8]. As a result, regulating microbial dysbiosis and disordered metabolism using dietary supplements has been strongly advocated [9], particularly aiming to reduce the side effect of small molecular drugs and improve quality of life for IBD patients.

Dietary components play an essential role in shaping the composition and function of the gut microbiota [10]. The health benefits of dietary fibers, such as fructo-, manno-, and galacto-oligosaccharides, have been long recognized [11,12]. While the efficacies of prebiotics, such as resistant starch and inulin, have been extensively studied in experimental colitis models with promising results, few studies have progressed to large-scale human clinical trials [13]. Recently, a randomized placebo-controlled trial to assess if inulin plus fructo-oligosaccharides (1:1) can prevent relapses in ulcerative colitis (UC) patients has been registered in the ClinicalTrials.gov database (https://clinicaltrials.gov/ct2/show/NCT02865707). The trial is still ongoing and no results have been reported.

Recently, seaweeds, one of the most abundant components of marine vegetation, have gained ample scientific attention because they are considered as a rich source of oligosaccharides with unique structural characteristics [14]. However, the exploitation of seaweed-derived oligosaccharides as alternative therapeutics in IBD management is still in its infancy. One of such oligosaccharides, neoagarotetraose (NT), a hydrolytic product of agar by β-agarase, has been reported to possess many biological properties [15]. For example, the anti-inflammatory properties of NT via the inhibition of MAPK and the NF-κB pathways have been demonstrated in murine macrophages [16]. NT is also known to alleviate fatigue-induced stress by regulating microbial composition and SCFA production [17]. Further, the relative abundance of *Bifidobacterium* is significantly increased as a result of NT supplementation in mice treated with antibiotics [18]. Moreover, several animal models have been used to evaluate the prebiotic effect of neoagarooligosaccharides [19]. However, the modulatory effect of NT on gut health and intestinal inflammation has not been examined in an experimental colitis model. Moreover, the regulation of NT on disordered gut microbial metabolism has yet to be investigated. Without a full understanding of the mechanisms of action, clinical trials to examine the efficacy of NT in colitis management would not be feasible. In this study, we attempted to evaluate the effect of NT in restoring microbial dysbiosis and alleviating dysregulated gut metabolome using a dextran sulfate sodium (DSS)-induced mice colitis model.

## 2. Materials and Methods

### 2.1. Animals Experiment

Twenty four C57BL/6J male mice (5–6 weeks old) were purchased from Charles River Laboratories (Wilmington, MA, USA). All animal care procedures were performed according to a protocol approved by the USDA Beltsville Area Institutional Animal Care Committee (Animal Protocol# 18-006a). The Institutional Animal Care and Use Committee (IACUC) guidelines were strictly followed. All experimental procedures were carried out in accordance with the approved guidelines. Mice were individually housed in ventilated cages for 14 days under a 12 h light/dark cycle at 24 °C before the experiment. All mice were fed a standard laboratory rodent diet ad libitum (AIN-93M) with free access to distilled water. After acclimation, the mice were randomly divided into three groups: Normal controls (NC; *n* = 8); DSS-induced colitis (MD; *n* = 8); DSS colitis mice supplemented with NT (NT; *n* = 7; one mouse was excluded due to the bodyweight loss exceeding 20% during experiment). NT (molecular weight: 630.55) was prepared using an enzymatic method, as previously reported [20]. The animals in the NC and MD groups continued with the AIN-93M diet. The mice in the NT group were fed AIN-93M containing NT at 900 mg per kg diet. The dietary trial started nine days prior to DSS induction and lasted through the two DSS cycles for a total of 51 days. To induce a colitis model, the colitis grade DSS (MW = 36,000–50,000; CAS# 9011-18-1, MP Biomedicals, Irvine, CA, USA) was used. The animals in the MD and NT groups were subject to two cycles of DSS in drinking water. Each cycle consisted of seven days of 2.5% DSS followed by 14 days of fresh tap water. At the end of a 51-day trial, the animals were sacrificed using the cardiac puncture technique, as approved in the animal protocol. The distal colon tissue samples were fixed in 10% neutral buffered formalin (NBF, Sigma; St Louis, MO, USA) for histology and the proximal colon tissue samples were used for RNA isolation. The entire colon contents were collected and mixed for microbiome and global metabolome analyses.

### 2.2. Diet Information

NT was prepared using β-agarase isolated from *Agarivorans gilvus WH080* [20]. β-agarase cleaves the β-1,4 linkages of agarose, producing neoagarooligosaccharides (NAO), including neoagarobiose, NT and neoagarohexaose. Briefly, 0.25% low-melting agarose and 1600 units of β-agarase were mixed and incubated at 35 °C for 48 h. The hydrolysis solution was then heated in boiling water for 10 min and concentrated using a vacuum-rotary evaporation apparatus at 55 °C. Three volumes of absolute ethanol were mixed with the concentrate, and the supernatant was then collected after centrifugation at 10,000 rpm for 15 min at 4 °C. The supernatant was repeatedly concentrated to powder without moisture. The powder was then dissolved in ~3 mL ultrapure water and then purified by gel filtration using 0.5 mol/L NH_4_ HCO_3_ as an eluent. The purified NT (>98%) was detected by thin-layer chromatography. Both AIN-93M and AIN-93M-containing NT at 900 mg per kg were formulated by Research Diets (New Brunswick, NJ, USA). The dose level was determined based on a previously published study [17] as well as a pilot experiment. The diet was sealed under vacuum and stored at −30 °C. The diet was changed every other day and the leftovers were weighed and recorded. The body weight (BW) was recorded daily. During the acclimation period, the mice in the NT group had an estimated mean ingestion of NT of 150.0 mg/kg BW/day. During the two DSS cycles, the mean ingestion of NT was 115.0 mg/kg BW/day, due to reduced feed intake.

### 2.3. Tissue Histology

The colon tissue samples fixed in 10% NBF were sectioned at 5 µm thickness and stained with hematoxylin and eosin. Tissue pathology was scored as follows: surface epithelial cells (0–4); edema (0–2); hemorrhage (0–2); crypt dilation (0–2); thickness of smooth muscle layer (0–3); number of inflammatory infiltrates (0–3), as previously published [21].

### 2.4. Gene Expression Analysis Using Quantitative RT PCR

Total RNA was extracted using TRIzol reagents (Invitrogen, Carlsbad, CA, USA) according to the manufacturer’s instructions. The crude total RNA was then purified using DNase digestion and a Qiagen RNeasy Micro Kit. The RNA concentration and integrity were measured using Nanodrop ND-1000 spectrophotometer (Thermo Scientific, Wilmington, MA, USA) and a BioAnalyzer 2100 (Agilent, Palo Alto, CA, USA), respectively. The cDNA was synthesized from purified total RNA using an iScript Advanced cDNA Synthesis Kit (BioRad, Hercules, CA, USA) and qRT-PCR reactions were performed in a CFX Connect Real-Time PCR Detection System (BioRad, Hercules, CA, USA). The reactions were run in duplicates in a total volume of 22 μL containing the following: 2 μL cDNA (100 ng); 0.5 μL of each primer (forward and reverse, 20 nM each); 11 μL SsoAdvanced Universal SYBR Green Supermix (BioRad, Hercules, CA, USA); 8 μL nuclease-free water. The amplification reactions were subject to an initial denaturation at 95 °C for 5 min, followed by 40 cycles of 95 °C for 30 s, 60 °C for 30 s, and 72 °C for 30 s. A standard curve-based absolute quantification method was used.

### 2.5. DNA Extraction and 16S rRNA Gene Sequencing

Total DNA was extracted from gut contents using a QIAamp PowerFecal DNA Kit according to the manufacturer’s instructions (Qiagen, Germantown, MD, USA). The hypervariable V3–V4 regions of the 16S rRNA gene amplification and sequencing were performed as previously described [3]. The primer sequences were as follows: forward primer, 341/357F, CCTACGGGNGGCWGCAG; reverse primer, 805/785R, GACTACHVGGGTATCTAATCC. A total of 40 ng DNA samples was amplified for 20 cycles. The amplified products from individual samples were purified using Agencourt AMPure XP beads (Beckman Coulter, Danvers, MA, USA). The purified PCR products were quantified using BioAnalyzer 2100 DNA 7500 chips and pooled based on an equal molar ratio and their respective samples-specific barcodes. The pooled libraries were sequenced using an Illumina MiSeq Reagent Kit v3 (2 × 255 cycles), as described previously [22]. The mean numbers of raw sequences were obtained, 154,027.87 ± 56,282.01 per sample. All raw sequence data were deposited to the NCBI SRA database (Accession# PRJNA637629).

### 2.6. Bioinformatic Analysis of 16S rRNA Gene Sequences

The quality of raw sequences was checked using FastQC v. 0.11.2. The sequences with low quality scores and the four maximally degenerate bases (NNNN) at the most 5’end of the read pair were removed using Trimmomatic v. 0.38. The processed pair-end reads were then merged using join_paired_ends.py. The parameter settings used were: the minimum overlap length was 20 bp and the maximum allowed mismatches within the overlapping region was 5%. The QIIME pipeline (v. 1.9.1) with the default reference v. 0.1.3 was used to analyze the 16S rRNA gene sequences. The “Closed reference” pipeline was used for picking operational taxonomic units (OTU)—pick_closed_reference_otus.py. The taxonomy assignment was based on the GreenGene database (v. 13.8). The rarefaction depth was set to 99,251 quality sequences per sample. Alpha diversity was extracted at the OTU level by the alpha_diversity.py procedure. Beta diversity was analyzed by the Primer software (v. 7.0), and the vegan package in R. PICRUSt (v. 1.1.2) was used to predict metagenome functional contents from marker gene (e.g., 16S rRNA) surveys with default parameters based on the OTU table generated using the closed-reference protocol in QIIME1. The significantly different features (taxa) between experimental groups were identified using a linear discriminant analysis (LDA) effect size (LEfSe) algorithm [23] with a default cutoff (the absolute log_10_ LDA score > 2.0 and *p* value < 0.05, based on Wilcoxon Rank Sum testing). In addition, for analysis of the composition of microbiomes (ANCOM [24]), a novel algorithm designed to handle the compositionality issue in marker gene count data was used to detect taxa differing significantly in two or more populations. ANCOM has been shown to be able to better control the false discovery rate (FDR) while improving power [24,25].

Microbial signatures or balances were identified using selbal (R v. 3.6.1) with default parameters [22]. Both Random Forest (RF) classification and regression models were performed using RandomForest R package (v. 4.6–14) [25]. The OTU table was first collapsed to a genus level count table. The abundance data were then transformed based on total sum scaling. The RF parameters used were as follows: the number of trees in the forest (ntree) was set to 501 and the number of features randomly sampled at each node in a tree (mtry) was 11. The Z-score, or scaled mean decrease accuracy, was calculated and used to rank feature or variable importance.

### 2.7. Global Metabolite Analysis

A Microlab STAR liquid handling system (Hamilton Company, Reno, Nevada, USA) was used for detecting metabolites in colon contents. The proteins in colon content samples were removed using methanol. Samples were placed briefly on a TurboVap (Zymark, Hopkinton, MA, USA) to remove the organic solvent. The samples were characterized using Ultrahigh Performance Liquid Chromatography–Tandem Mass Spectroscopy (UPLC–MS/MS) consisting of a Waters ACQUITY ultra-performance liquid chromatograph (UPLC) and a Thermo Scientific Q-Exactive high resolution/accurate mass spectrometer, interfaced with a heated electrospray ionization (HESI-II) source and Orbitrap mass analyzer, operated at 35,000 mass resolutions. Raw data were extracted, peaks were identified, and then quantified using the area under the curve method. Each compound was corrected in run/day blocks. The peak intensity data were normalized based on the median and log transformed. Normalized ion count data were analyzed using a Wilcoxon Rank Sum test to identify metabolites that may differ significantly between experimental groups.

### 2.8. Statistical Analysis

Where applicable, statistical testing was performed by the Wilcoxon rank sum test (Mann–Whitney U test), which does not assume normality, using the R function wilcox.test (R v. 3.6.1; https://cran.r-project.org). Differences were considered significant when *p* < 0.05. In addition, for analysis of the composition of microbiomes, ANCOM v. 2.1 was used to detect taxa differing significantly between two populations. Microbial signatures or balances were identified using selbal (https://github.com/UVic-omics/selbal) with default parameters. Random Forest (RF) classification and regression models were performed using RandomForest R package (v. 4.6–14).

## 3. Results

### 3.1. Neoagarotetraose Promoted the Repair of DSS-Induced Colon Tissue Damage

**Figure 1:**
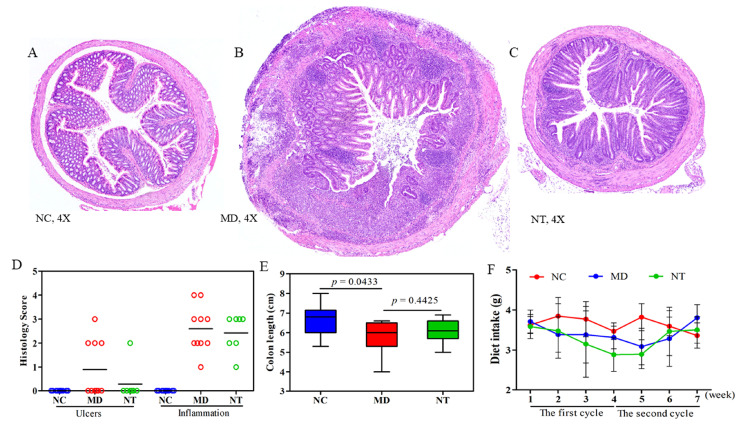
Neoagarotetraose (NT) promoted mucosal healing in dextran sulfate sodium (DSS)-induced murine colitis mice. Hematoxylin and Eosin staining of (**A**) normal controls (NC), (**B**) colitis induced by DSS (MD), and (**C**) colitis mice supplemented with NT. (**D**) Historical scores of ulceration and the number of inflammatory infiltrates was improved due to NT supplementation. (**E**) The colon length was significantly reduced by DSS. (**F**) Daily feed intake during the two DSS cycles.

DSS induced a marked change in the morphology and organization of the colon mucosa, as shown in Figure 1. Compared with NC, shown in Figure 1A, the colon muscle layer in DSS colitis mice (MD) was significantly thicker, and the severity of ulceration and crypt dilation was more profound, as shown in Figure 1B, C). The number of inflammatory infiltrates was also significantly increased, as shown in Figure 1D. The colon length was significantly shortened by DSS (*p* < 0.05). The mice treated with DSS also had a significant loss in body weight, as shown in Supplementary Figure S1A (*p* < 0.05), while NT supplementation did not appear to have a significant effect on the colon length, as shown in Figure 1E, or body weight. Moreover, an improvement in the morphology of the colon mucosa in the colitis mice supplemented with NT was notable, as shown in Figure 1C. Compared to the MD group, NT significantly reduced the score of ulceration and the number of inflammatory infiltrates, as shown in Figure 1D. The difference in daily feed intake was insignificant between the MD and NT groups, as shown in Figure 1F.

The gene expressions of IL1β, IL4, IL5, IL6, IL17, iNOS, NOD2, TLR4, TLR9 and TNF in the colon tissue were evaluated using qRT-PCR, as shown in Supplementary Table S1. While DSS induced a significant increase in the mRNA level of multiple genes, including IL17, iNOS, and TNF, comparing to normal controls, as shown in Supplementary Figure S1, NT ingestion had no significant effects on the gene expression levels of all ten genes examined.

### 3.2. Neoagarotetraose Partially Restored Gut Microbial Dysbiosis Induced by DSS

**Figure 2:**
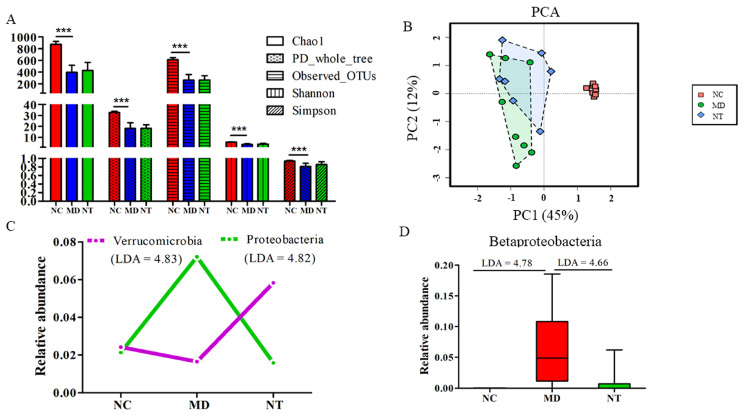
The modulatory effect of neoagarotetraose (NT) on the murine gut microbiome. NC: normal control mice. MD: DSS induced colitis mice. NT: DSS induced colitis mice supplemented with NT. (**A**) Alpha diversity indices impacted by DSS and NT supplementation. *** *p* < 0.01 based on Wilcoxon Rank Sum testing. (**B**) Beta diversity based on Principal component analysis (PCA). (**C**) The relative abundance of select phyla. Absolute log_10_ Linear Discriminant Analysis (LDA) scores were obtained using the LEfSE algorithm. (**D**) The relative abundance of the class β Proteobacteria significantly reduced by NT.

Microbial alpha diversity indices, including Chao1, phylogenetic diversity (PD whole tree), the number of observed operational taxonomic units (OTU), Shannon and Simpson, were significantly reduced by DSS, as shown in Figure 2A, (*p* < 0.001). For example, Chao1 was significantly repressed by DSS, from 822.55 ± 65.05 in NC to 397.29 ± 120.09 in the MD group (*p* = 3.48 × 10^−7^). DSS also had a significant impact on beta diversity, as shown in Figure 2B. Principal component analysis (PCA), based on Bray–Curtis similarities, shows that the samples in the NC group were clearly separated from those treated with DSS, with or without NT supplementation. The result from the Analysis of Similarities (ANOSIM) also indicated that the microbial community of the colitis mice, induced by DSS, was distinctly different from those in normal controls *p* = 0.001, as shown in Supplementary Figure S2. DSS had a significant effect on the gut microbial composition, affecting the two most abundant phyla, *Bacteroidetes* (absolute log_10_ linear discriminant analysis (LDA) = 4.79) and *Firmicutes* (LDA = 4.81). The relative abundance of *Bacteroidetes* was increased from 46.13% in normal mice to 69.39% in colitis mice (MD) with a concurrent decrease in the relative abundance of *Firmicutes*, from 47.31% in NC to 20.02% in MD. At the class level, the relative abundance of *Betaproteobacteria* in colitis mice was significantly higher than in normal mice, as detected by both LEfSE and ANCOM algorithms. At the species level, the abundance of *Clostridium difficile* and *C. perfringens* was significantly increased by ~739-fold and ~1678-fold in the colitis mice, compared to in the normal controls, respectively (data not shown).

NT supplementation did not appear to have a significant effect on microbial alpha diversity, as shown in Figure 2A. The values for Chao1, PD_whole_tree, Observed OTUs, Shannon and Simpson were marginally increased by NT supplementation but did not reach a statistically significant level. ANOSIM also showed that there was no significant separation between the NT and MD groups in beta diversity, as shown in Supplementary Figure S2. Nevertheless, NT ingestion had a significant effect on the abundance of at least two phyla, Proteobacteria and Verrucomicrobia, as shown in Figure 2C. The abundance of Verrucomicrobia was increased from 1.64% in the MD group to 5.82% in the NT group. On the other hand, the relative abundance of Proteobacteria was significantly decreased from 7.20% in the MD group to 1.57% in the NT group, a level close to what was observed in the normal controls. Notably, NT reversed the elevated level of the class Betaproteobacteria in the MD group to the base line in normal controls, as shown in Figure 2D (based on both LEfSE and ANCOM).

**Figure 3:**
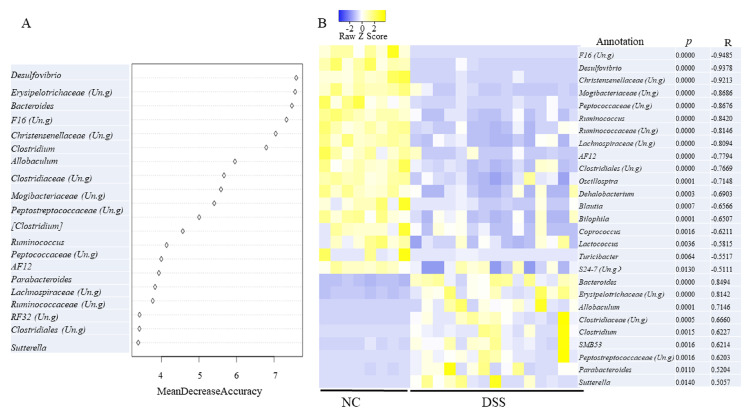
Microbial genera significantly related to DSS induced colitis phenotypes. (**A**) The Random Forest analysis identified important microbial genera distinguishing colitis phenotypes. Un.g: unclassified genus in a given family. (**B**) Taxa significantly correlated with the colitis status. P: significance; R: correlation coefficient.

**Figure 4:**
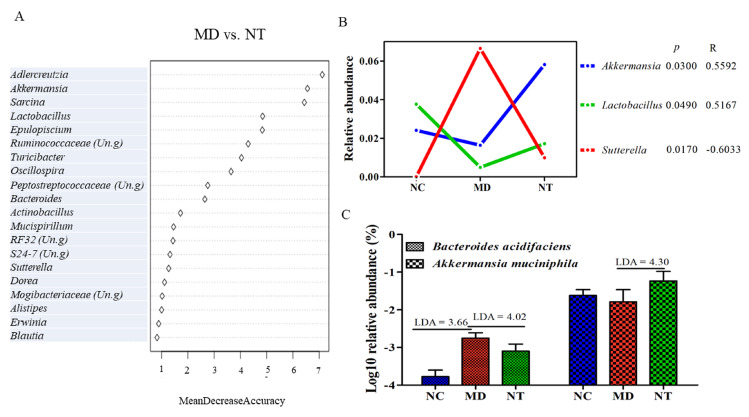
Bacterial species and genera significantly impacted by neoagarotetraose (NT). (**A**) The most important variables (genera) identified by Random Forest analysis that were discriminative of NT supplementation status. (**B**) The relative abundance of three genera significantly affected by NT. (**C**) Bacterial species significantly impacted by NT supplementation. P: significance; R: correlation coefficient. NC: normal control mice. MD: DSS induced colitis mice. NT: DSS induced colitis mice supplemented with NT.

The Random Forest (RF) algorithm, one of popular machine learning tools, was used to estimate the correlation between environmental variables and important microbial taxa. As Figure 3A shows, the top 20 genera provided an excellent accuracy in classifying NC and MD. The genus *Desulfovibrio*, ranked as the most important feature, based on mean decrease accuracy, was found to be 6.19-fold more abundant in NC than in colitis mice (MD). Moreover, a Pearson correlation analysis between the status of the DSS treatment and the relative abundance of various genera was performed. An unclassified genus in the family F16 and *Desulfovibrio* had a strong, yet negative, correlation with the DSS status, as shown in Figure 3B (R < −0.93, *p* < 0.05). An unclassified genus in the families Christensenellaceae was also strongly and negatively correlated with the DSS status (*p* < 0.0000; R < −0.92). The latter family is implicated in multiple human diseases, including IBD [26]. On the other hand, *Bacteroides* was positively correlated with DSS treatment (R = 0.8494, *p* < 0.05) and was 11.32-fold more abundant in DSS treated mice than in the normal controls. This genus was also among the most important variables with respect to classifying the DSS status. The abundance of *Sutterella* was increased from 0.002 in NC to 6.66% in colitis mice (MD). Furthermore, *Sutterella* was ranked as the most important genera in contributing to classifying the NT supplementation status, as shown in Figure 4A. Intriguingly, the abundance of *Sutterella* was significantly decreased by NT ingestion, as shown in Figure 4B (LDA = 4.47), and it was negatively correlated with NT ingestion, as shown in Figure 4B (*p* < 0.05; R = −0.60). In addition, the abundance of *Lactobacillus* and *Akkermansia* was significantly higher in the NT group than in the MD group, and both were significantly correlated with the NT supplementation status, as shown in Figure 4B. These two genera were also among the most important genera in contributing to the classification accuracy. At the species level, NT supplementation resulted in a significant increase in the relative abundance of *Akkermansia muciniphila,* as shown in Figure 4C.

### 3.3. Microbial Signatures for Neoagarotetraose Supplementation

**Figure 5:**
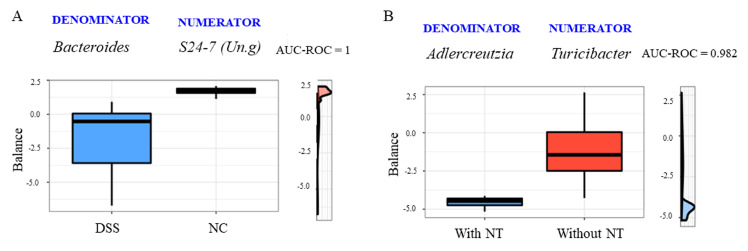
Microbial signatures or global balances selected by the selbal algorithm with a strong accuracy to distinguish colitis phenotypes (**A**) or neoagarotetraose (NT) supplementation status (**B**). AUC = Area under the ROC (Receiver Operating Characteristics) curve. The box plots represent the distribution of the balance values for each category. The right (vertical) panel of the figure represents density curves for each category. NC: normal controls; B: colitis induced by dextran sulfate sodium.

Microbial signatures with predictive accuracy for the colitis and NT supplementation status were identified using selbal. The signature or balance, consisting of *Bacteroides* (denominator) and an unclassified genus (Un.g) in the family S24-7 (numerator), had an area under the receiver operating characteristic curve (AUC) of 1.00 for the DSS-induced colitis condition (mean cross validation or CV-AUC = 1.00), as shown in Figure 5A. The balance value was negative for the colitis phenotype, indicating that the relative abundance of the denominator, *Bacteroides*, was much higher than the nominator. Moreover, the signature represented by the log ratio of the abundance of *Adlercreutzia* (denominator) and *Turicibacter* (numerator) can robustly predict the NT supplementation status (CV-AUC = 0.79). The negative balance values for the NT group resulted from the significant decrease in the abundance of *Turicibacter* due to NT ingestion, as shown in Figure 5B.

### 3.4. Neoagarotetraose Reversed Key Metabolic Pathways Dysregulated by DSS 

**Figure 6:**
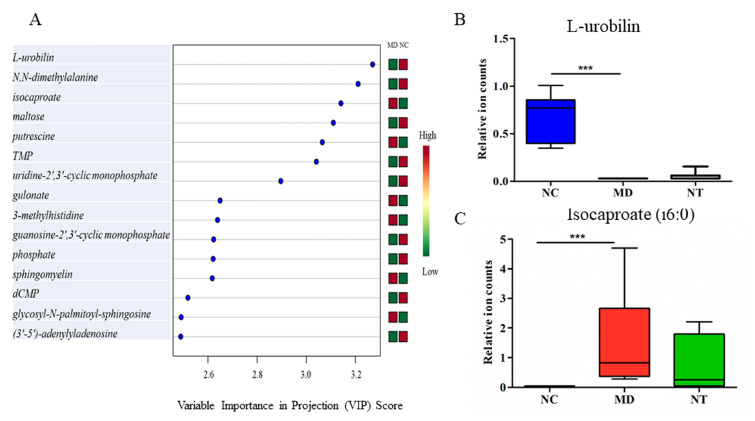
Urobilin related pathways dysregulated in a colitis murine model induced by dextran sulfate sodium. (**A**) Partial least squares discriminant analysis (PLS-DA) identified L-urobilin, N, N-dimethylalanine and isocaproate as the top three important metabolites that were discriminative of the colitis phenotype. (**B**) Gut luminal L-urobilin concentrations were significantly reduced by DSS. (**C**) Isocaproate levels were significantly higher in colitis mice. NC: normal controls; MD: colitis induced by DSS; NT: DSS induced colitis mice supplemented with NT. *** *p* < 0.001.

DSS induced a significant change in the gut metabolome in mice, in good agreement with the previous study [3]. Up to 55% of all metabolites detected in the gut contents were significantly dysregulated by DSS, leading to a clear separation in the PCA plot between normal controls and colitis groups, as shown in Supplementary Figure S3. These metabolites belong to a broad range of metabolic pathways, involved in the metabolism of amino acids, carbohydrates, cofactors and vitamins, energy, lipids, nucleotides, peptides and xenobiotics. Partial least squares discriminant analysis (PLS-DA) ranked L-urobilin, as shown in Supplementary Figure S4, N, N-dimethylalanine, isocaproate (i6:0), maltose and putrestine as the top five metabolites in distinguishing the DSS-induced colitis group from normal controls, as shown in Figure 6A. L-urobilin, assigned to the hemoglobin and porphyrin metabolism pathways, was significantly reduced by DSS (*p* < 0.001), as shown in Figure 6B. On the contrary, DSS led to a 73-fold increase in the level of isocaproate (*p* < 0.001), as shown in Figure 6C. Moreover, DSS had a profound impact on histidine metabolism, as shown in Supplementary Table S2. Among the 18 metabolites assigned to this pathway detected in this study, 12 were significantly increased by DSS. For example, 3-methylhistidine, which was elevated approximately 57-fold by DSS, was ranked as the most important feature in distinguishing the colitis status, as shown in Figure 6A.

**Figure 7:**
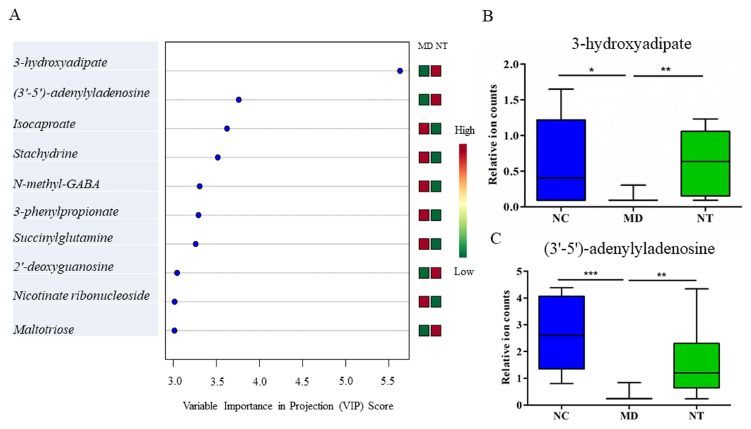
Important metabolites related to neoagarotetraose (NT) supplementation. (**A**) Metabolite predictors discriminative of NT supplementation status based on variable importance in projection scores. (**B**) NT supplementation significantly reversed the 3-hydroxyadipate concentrations from a repressed level in DSS induced colitis mice. (**C**) NT supplementation restored the (3’–5’)-adenylyladenosine repressed by DSS to the basal level observed in normal controls. NC: normal controls; MD: colitis induced by DSS; NT: DSS-induced colitis mice supplemented with NT. * *p* < 0.05; ** *p* < 0.01; *** *p* < 0.001.

NT supplementation had a notably modulatory effect on dysregulated metabolic pathways in colitis mice. Among the 37 metabolites affected by NT, approximately 70% (i.e., 26 metabolites were also dysregulated by DSS), as shown in Table 1. NT ingestion resulted in a significant increase in the level of cis-urocanate, compared to in the colitis mice (*p* < 0.05). Furthermore, a marginally but nevertheless significant increase in gut 4-imidazoleacetate levels was observed in the NT supplemented mice. Interestingly, the relative abundance of the microbial histidine metabolism pathway, as well as histidine kinase, displayed a significant increase in the MD group, as shown in Supplementary Figure S5 (*p* < 0.05), and this pathway had a strong correlation with DSS treatment (R = 0.8948, *p* < 0.001). Moreover, it was almost reversed to the basal level by NT, as shown in Supplementary Figure S5. 3-hydroxyadipate, 3’-5’-adenylyladenosine, isocaproate (i6:0), stachydrine and N-methyl-GABA were the top five metabolites in distinguishing NT supplementation status, as shown in Figure 7A. Both 3-hydroxyadipate and 3’-5’-adenylyladenosine were significantly decreased by DSS. Moreover, NT significantly reversed these metabolites to their base line levels, as shown in Figure 7B,C.

**Figure 8:**
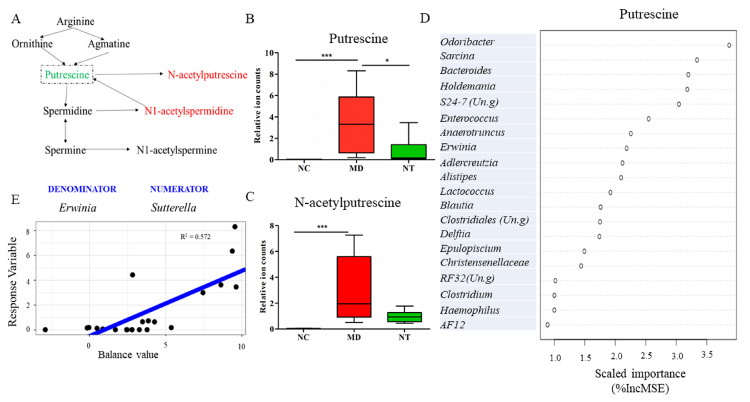
Microbial signatures correlated with gut luminal putrescine levels. (**A**) A diagram showing the arginine metabolism pathway. (**B**) The levels of putrescine and N-acetylputrescine (**C**) significantly impacted by neoagarotetraose (NT). (**D**) Microbial genera that were correlated with gut putrescine concentrations identified using a Random Forest regression model. E: A global balance or microbial signature strongly correlated with gut putrescine concentrations. NC: normal controls; MD: colitis induced by DSS; NT: DSS induced colitis mice supplemented with NT. %IncMSE: Percentage increase in mean squared error. * *p* < 0.05; *** *p* < 0.001.

The observation that several metabolites related to polyamine metabolism were altered by DSS prompted us to investigate the potential inhibitory effect of NT on putrescine. As Figure 8A shows, arginine gets degraded into ornithine and agmatine, and these two metabolites are then converted into putrescine. DSS induced a 255-fold increase in gut luminal putrescine concentration (*p* = 0.0000), compared to normal controls, as shown in Figure 8B. The colitis mice supplemented with NT had a significantly decreased putrescine level (*p* < 0.05), as shown in Figure 8B. N-acetylputrescine, a product of putrescine, was concomitantly increased by DSS, as shown in Figure 8C (*p* < 0.001). The RF regression model suggested that *Odoribacter, Sarcina* and *Bacteroides* were the top microbial predictors for putrescine levels, as shown in Figure 8D. In addition, a global balance consisting of *Erwinia* (denominator) and *Sutterella* (numerator) displayed a good correlation with putrescine levels (R^2^ = 0.572), as shown in Figure 8E. 

## 4. Discussion

Dietary fiber is a popular prebiotic due to its potential health benefits [27]. With a rapid increase in world population and subsequent high demand for food, the sustainable production of agriculturally-derived dietary fiber is facing big challenges due to limited land availability. Marine algae, with the properties of easy cultivation and the ready production of bioactive compounds and high contents of dietary fiber (about 33~75% dry weight), have attracted sufficient scientific interest in recent years [28]. Moreover, as the most promising components of dietary fiber, polysaccharides and oligosaccharides derived from marine algae contain unique substitutions, such as sulfation or carboxylation [29], which may possess different activities for human health, unlike those commonly found in fruits and vegetables. As a result, an increasing number of novel marine-derived polysaccharides and oligosaccharides is being discovered and their potential health-promoting properties are being recognized [30,31,32]. NT, the predominant member of the neoagarooligosaccharides that can inhibit bacterial growth and stimulate macrophages [15], is known to possess properties to modulate intestinal microbiome [17]. However, the potential prebiotic effects of NT on experimental colitis model IBD are still unclear. In this study, we attempted to understand the molecular mechanisms of NT in modulating gut microbiome and metabolome and alleviating intestinal inflammation. 

The dysregulated resolution of inflammation is one of the critical factors in IBD pathogenesis [33]. The clinical outcome of mucosal healing is considered as one of the mechanisms for the proper resolution of inflammation in IBD [34]. Our data show that the DSS-induced colon structure damage and the histology score of ulceration and inflammation were improved as a result of 51-day NT ingestion. This finding suggests that NT may be implicated in regulating the resolution of inflammation. While the expression levels of pro-inflammatory cytokines and modulators, such as IL1**β**, IL17, iNOS and TNF, were significantly increased in the DSS-induced colitis model, our data show that NT ingestion did not appear to have a direct effect on the cytokines involved in the onset (initiation phase) of inflammation. This observation prompted us to search for other molecular mechanisms through which NT regulated intestinal inflammation. 

Luminal bacteria play a critical role in intestinal mucosa healing [2,35]. In this study, DSS induced a significant reduction in microbial alpha diversity, such as Chao1, Shannon, Simpson and PD whole trees, as shown in Figure 2A (*p* < 0.001), in a good agreement with previous studies [3,36]. NT supplementation resulted in slight improvements in the alpha diversity. However, the impact of NT on gut microbial composition was more profound. In general, an elevated level of Proteobacteria and a decreased Firmicutes contributed to the unbalanced composition of gut microbiota [37]. In our study, the relative abundance of Firmicutes was significantly decreased from 47.31% in the normal controls to 20.02% in the colitis mice (LDA = 4.81), while the abundance of Proteobacteria was increased from 2.13% to 7.20% by DSS. The members of Proteobacteria, a rich source of lipopolysaccharides (LPS), are an important factor in contributing to mucosal inflammation [38]. NT ingestion completely reversed the relative abundance of Proteobacteria from an elevated level in colitis mice (7.20%) to 1.57%, as shown in Figure 2C (LDA = 4.83). At the class level, the abundance of β-proteobacteria was significantly decreased by ~ 7-fold due to NT supplementation, as shown in Figure 2D (LDA = 4.66). Furthermore, NT ingestion significantly increased the abundance of the phylum Verrucomicrobia (LDA = 4.82). Notably, one of its key members, *Akkermansia muciniphila*, was significantly increased as a result of NT ingestion, as shown in Figure 4C (*p* < 0.05). Intriguingly, *Akkermansia*, as a member of the wound-associated consortium, is significantly increased during wound repairs. *Akkermansia muciniphila*-induced enhancement in mucosal wound repair involves intestinal epithelial cell-specific neutrophilic NADPH oxidase (NOX1)-dependent redox signaling [39]. An animal study using a DSS-induced mouse model demonstrates that *A. muciniphila* treatment is negatively associated with pro-inflammatory cytokine expression [40]. Our study identified *Akkermansia* as one of the most important microbial features distinguishing the NT supplementation status, providing further support to its critical role in ameliorating mucosal healing. In addition, the abundance of *Bacteroides acidifaciens* is frequently elevated during inflammation followed by a rapid post-colitis decrease [41]. In our study, *Bacteroides* was the key member of a microbial signature with an accurate predictive power for the colitis phenotype. RF analysis also ranked this genus as the most important feature distinguishing DSS-induced colitis from healthy mice. Many members of *Bacteroides*, such as *B. fragilis*, *B. ovatus,* and *B. acidifaciens,* play important roles in IBD pathogenesis [42]. Together, our findings suggest that NT may be involved in promoting the proper resolution of intestinal inflammation by regulating gut microbial composition.

Gut metabolites have a strong correlation with intestinal microbial composition and gut mucosa healing [43,44]. Our data showed that DSS significantly altered a total of 403 metabolites that are involved in a broad spectrum of pathways. The alterations of amino acid pathways in IBD patients have been reported [45]. In our study, the histidine metabolic pathway was profoundly impacted by DSS. Histidine is converted into urocanate via histidine ammonia-lyase (HAL) or histamine via L-histidine decarboxylase (HDC). Histamine, a mediator of inflammation [46], was significantly elevated ~25-fold by DSS. Moreover, NT ingestion resulted in a significant change in at least 37 metabolites, including amino acids, lipids, carbohydrate, cofactors and vitamins, nucleotides and xenobiotics. For example, NT ingestion led to a significant increase in gut luminal cis-urocanate level and a concurrent decrease in histamine levels. The involvement of cis-urocanate in promoting the expression of IL10 mRNA and protein in murine spleen cells has been documented [47]. In our model, the mRNA expression of IL10 was marginally elevated by NT. Taken together, our findings suggest that NT may tilt the histidine metabolism towards urocanate conversion, away from histamine biosynthesis, contributing to its anti-inflammatory properties. Furthermore, the metabolites in the polyamine metabolic pathway were also impacted by NT supplementation. Polyamines, such as putrescine, spermidine and spermine, have a relatively high concentration in rapidly growing tissues, and their contents are rapidly increasing during tissue proliferation [48]. Our results show that DSS significantly increased the concentration of putrescine and N-acetylputrescine, while NT ingestion significantly decreased gut luminal putrescine levels. The decreased putrescine level may directly contribute to colon wound healing in NT supplemented mice. In addition, the production of polyamines is closely related to gut bacteria, particularly those in the genus *Bacteroides,* as well as *Escherichia coli* and *Enterococcus faecalis* [49]. Indeed, our RF regression models identified *Sutterella*, *Odoribacter, RF32 (Un.g)*, *Oscillospira* and *Enterobacteriaceae (Un.g)* as the most important features correlated with gut luminal putrescine levels, as shown in Figure 8D. A microbial signature, consisting of *Erwinia* (denominator) and *Sutterella* (numerator), also had a positive association with putrescine levels, as shown in Figure 8E. *Sutterella*, a member of the phylum Proteobacteria, is more abundant in IBD, autism, and obesity [50,51]. Therefore, *Sutterella* can be used as a predictor or biomarker for luminal putrescine levels and subsequent gut mucosal healing. 

Host-derived metabolites in tocopherol metabolism were partially restored by NT from the repressed levels induced by DSS, as shown in Supplementary Table S3. For example, delta-tocopherol was decreased 2.32-fold by DSS, while its content was increased by 1.74-fold by NT ingestion. The change of alpha-tocopherol and gamma-tocopherol/beta-tocopherol exhibited a similar trend. All four tocopherols belong to the vitamin E family. Moreover, gamma-tocopherol/beta-tocopherol and delta-tocopherol possess unique antioxidant and anti-inflammatory properties, by suppressing the NF-κB and STAT3/6 pathways [52]. Further, the pathogenicity of bacteria in the murine intestinal tract is exacerbated under vitamin E deficiency [21].

The metabolite signatures identified by RF can be readily used to discriminate the colitis status and may be developed as biomarkers. L-urobilin, for example, was ranked as the most important metabolite in discriminating healthy and DSS-induced colitis phenotypes, as shown in Figure 6A. In good agreement with human results, the level of L-urobilin was significant reduced in DSS-induced colitis [53]. On the other hand, 3-hydroxyadipate was the most important feature to discriminate NT supplementation status. 

The findings presented in this study demonstrated that NT was able to mitigate the pathology and severity of phenotypes in an experimental colitis model and provided novel insights into its mechanism of action. However, limitations of the current study included the small sample size and lack of dose-dependent effects. The difference in the route of NT administration should be considered as well. Moreover, while the modulatory effect of NT on pro-inflammatory cytokine expression in the gross colon tissue was insignificant, its role on individual immune cells may be different. Our future work will target the regulatory effect of NT on various immune cells using a single-cell transcriptome approach. Nevertheless, the molecular and microbial signatures identified should facilitate the development of NT as a potent prebiotic for promoting mucosal healing. 

## 5. Conclusions

Neoagarooligosaccharodes (NAO) are known to possess bioactive properties, such as the inhibition of bacterial growth, stimulation of macrophages and natural killer cells and the enhancement of antitumor immunity. As a predominant member of NAO, NT is able to alleviate fatigue stress by regulating fecal microbial composition and short chain fatty acid production. In this study, we demonstrated that NT improved gut integrity and inflammation scores in a DSS–induced murine colitis model. NT partially restored DSS-induced gut dysbiosis. Further, NT reversed the abundance of Proteobacteria from an elevated level in colitis mice and significantly increased the abundance of Verrucomicrobia, particularly those beneficial bacteria, such as *Akkermansia* as well as *Lactobacillus*. Moreover, NT significantly modulated multiple gut metabolites, particularly those related to histidine, polyamine and tocopherol metabolic pathways. The metabolite and microbial signatures identified had high prediction accuracy for the severity of DSS-induced colitis phenotypes and the NT supplementation status and can be developed as biomarkers. Our findings provided novel insights into the mechanisms by which NT modulated the gut microbiome and metabolome and should facilitate the optimization of NT as a potent prebiotic for colitis management. 

## Figures and Tables

**Table 1:** Gut metabolites significantly affected by neoagarotetraose supplementation. *P* value was calculated based on Wilcoxon Rank Sum testing. Fold was defined as the ratio of mean normalized ion counts of MD to NC or NT to MD. NC: normal baseline controls; MD: colitis induced by DSS; NT: DSS induced colitis mice supplemented with neoagarotetraose.

		*p* Value		Fold		
Pathway	Metabolite	MD/NC	NT/MD	MD/NC	NT/MD	Category
Aminosugar Metabolism	fucose	0.0213	0.0244	2.73	0.35	Carbohydrate
Aminosugar Metabolism	N-acetylglucosamine 6-sulfate	0.0003	0.0273	9.15	0.26	Carbohydrate
Ascorbate and Aldarate Metabolism	gulonate	0.0021	0.0413	60.57	0.06	Cofactors and Vitamins
Creatine Metabolism	guanidinoacetate	0.0585	0.0411	1.86	0.49	Amino Acid
Dinucleotide	(3’-5’)-adenylyladenosine	0.0000	0.0158	0.12	5.14	Nucleotide
Fatty Acid Metabolism	butyrylglycine	0.0178	0.0178	1.86	0.54	Lipid
Fatty Acid, Dicarboxylate	2-hydroxyglutarate	0.0152	0.0058	1.81	0.45	Lipid
Fatty Acid, Dicarboxylate	3-hydroxyadipate	0.0107	0.0270	0.22	4.19	Lipid
Fatty Acid, Dicarboxylate	dodecadienoate (12:2)	0.0003	0.0161	4.82	0.27	Lipid
Fatty Acid, Dicarboxylate	suberate (C8-DC)	0.0488	0.0307	0.63	0.59	Lipid
Fatty Acid, Monohydroxy	3-hydroxydecanoate	0.0001	0.0157	4.68	0.37	Lipid
Food Component/Plant	dipicolinate	0.2604	0.0159	1.51	0.24	Xenobiotics
Food Component/Plant	piperidine	0.3545	0.0102	1.49	0.27	Xenobiotics
Food Component/Plant	vanillate	0.0069	0.0082	1.43	0.71	Xenobiotics
Galactosyl Glycerolipids	1-palmitoyl-galactosylglycerol (16:0)	0.0116	0.0012	0.47	3.02	Lipid
Glutamate Metabolism	S-1-pyrroline-5-carboxylate	0.0009	0.0462	2.88	0.53	Amino Acid
Glutamate Metabolism	succinylglutamine	0.0002	0.0181	24.30	0.18	Amino Acid
Glycine, Serine and Threonine Metabolism	N-acetylserine	0.0001	0.0291	3.82	0.40	Amino Acid
Histidine Metabolism	cis-urocanate	0.1436	0.0074	1.65	2.16	Amino Acid
Long Chain Monounsaturated Fatty Acid	erucate (22:1n9)	0.8395	0.0335	1.01	1.90	Lipid
Lysine Metabolism	N-acetyl-cadaverine	0.1339	0.0025	2.06	2.12	Amino Acid
Lysophospholipid	1-oleoyl-GPG (18:1)	0.0119	0.0449	3.92	2.51	Lipid
Medium Chain Fatty Acid	caprylate (8:0)	0.0750	0.0156	1.52	0.54	Lipid
Medium Chain Fatty Acid	cis-4-decenoate (10:1n6)	0.0049	0.0224	4.09	0.30	Lipid
Medium Chain Fatty Acid	heptanoate (7:0)	0.0017	0.0379	2.53	0.43	Lipid
Nicotinate and Nicotinamide Metabolism	nicotinamide riboside	0.7553	0.0289	1.44	2.42	Cofactors and Vitamins
Phosphatidylethanolamine (PE)	1-oleoyl-2-linoleoyl-GPE (18:1/18:2)	0.0309	0.0467	4.58	0.21	Lipid
Phosphatidylethanolamine (PE)	1-palmitoyl-2-linoleoyl-GPE (16:0/18:2)	0.0011	0.0456	2.89	0.52	Lipid
Phospholipid Metabolism	glycerophosphoserine	0.0000	0.0441	19.33	0.25	Lipid
Purine, (Hypo)Xanthine/Inosine containing	2’-deoxyinosine	0.0022	0.0051	0.34	3.57	Nucleotide
Purine Metabolism, Guanine containing	2’-deoxyguanosine	0.0025	0.0068	0.28	5.09	Nucleotide
Pyrimidine Metabolism, Uracil containing	beta-alanine	0.1512	0.0282	1.95	0.37	Nucleotide
Sterol	campesterol	0.3166	0.0169	0.91	1.38	Lipid
Sterol	stigmastadienone	0.0133	0.0001	0.87	1.34	Lipid
TCA Cycle	2-methylcitrate/homocitrate	0.0751	0.0447	1.59	0.55	Energy
Tocopherol Metabolism	delta-tocopherol	0.0021	0.0307	1.61	1.45	Cofactors and Vitamins
Tryptophan Metabolism	indole-3-carboxylate	0.0458	0.0145	1.68	0.52	Amino Acid

## Supplementary Materials

The following are available online at https://www.mdpi.com/2076-2607/8/7/995/s1, Figure S1. Bodyweight changes and gene expression in colon mucosa Changes in mean body weight change after two cycle DSS treatment (A). The mRNA expression of TNF (B), IL17 (C) and iNOS (D) in colon mucosal tissue. NC: Normal controls; MD: DSS-induced colitis mice; NT: the DSS-induced colitis mice supplemented with a daily dose of 150 mg/kg bodyweight of neoagarotetraose for a total of 51 days, Figure S2. Analysis of similarities (ANOSIM) of beta diversity. NC: Normal controls; MD: DSS-induced colitis mice; NT: the DSS-induced colitis mice supplemented with NT, Figure S3. A Principal component analysis (PCA) plot based on global metabolite data. NC: Normal controls; MD: DSS-induced colitis mice, Figure S4. A diagram showing the L-Urobilin metabolism pathway, Figure S5. The microbial histidine kinase and histidine metabolism pathway significantly affected by neoagarotetraose supplementation NC: Normal controls; MD: DSS-induced colitis mice; NT: the DSS-induced colitis mice supplemented with a daily dose of 150 mg/kg bodyweight of neoagarotetraose for a total of 51 days, Table S1: Primers used in the study, Table S2. Select metabolites related to histidine metabolism were dysregulated by DSS. NC: normal controls; MD: colitis induced by DSS; NT: DSS induced colitis mice supplemented with NT, Table S3. Select metabolites belonging to tocopherol metabolism were significantly affected by NT supplementation. NC: normal controls; MD: colitis induced by DSS; NT: DSS induced colitis mice supplemented with NT.
