# Supplementary material for: Molecular and Microbial Signatures Predictive of Prebiotic Action of Neoagarotetraose in a Dextran Sulfate Sodium-Induced Murine Colitis Model
Source: Microorganisms. 2020 Jul 3;8(7):995. doi: 10.3390/microorganisms8070995 (PMC7409226; doi:10.3390/microorganisms8070995)
Supplement: Supplementary file 1 [file microorganisms-08-00995-s001.pdf]

Molecular and microbial signatures predictive of prebiotic action of neoagarotetraose in a dextran sulfate sodium-induced murine colitis model

Fang Liu, Jianan Liu, Thomas T.Y. Wang, Changhu Xue, Xiangzhao Mao*,

Qing-juan Tang*, Robert W. Li

Supplementary materials

Figure S1. Bodyweight changes and gene expression in colon mucosa. Changes in mean body weight change after two cycle DSS treatment (A). The mRNA expression of TNF (B), IL17 (C) and iNOS (D) in colon mucosal tissue. NC: Normal controls; MD: DSS-induced colitis mice; NT: the DSS-induced colitis mice supplemented with a daily dose of 150 mg/kg bodyweight of neoagarotetraose for a total of 51 days.

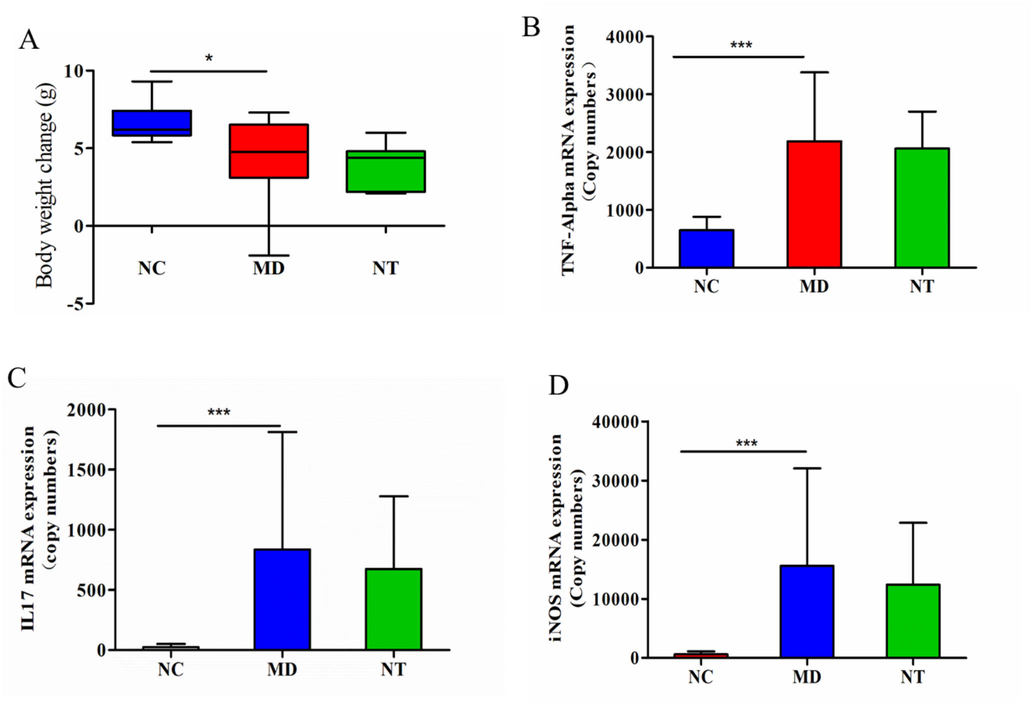

Figure S2. Analysis of similarities (ANOSIM) of beta diversity. NC: Normal controls; MD: DSS-induced colitis mice; NT: the DSS-induced colitis mice supplemented with NT.

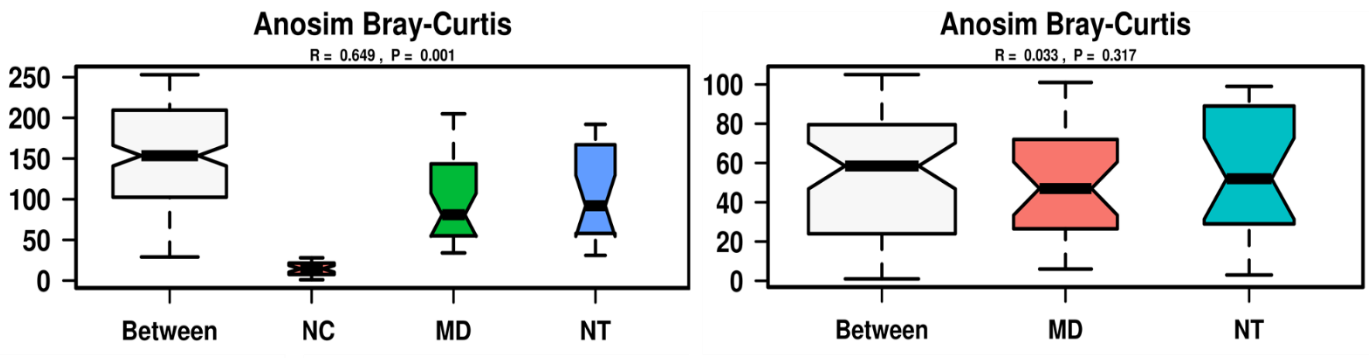

Figure S3. A Principal component analysis (PCA) plot based on global metabolite data. NC: Normal controls; MD: DSS-induced colitis mice.

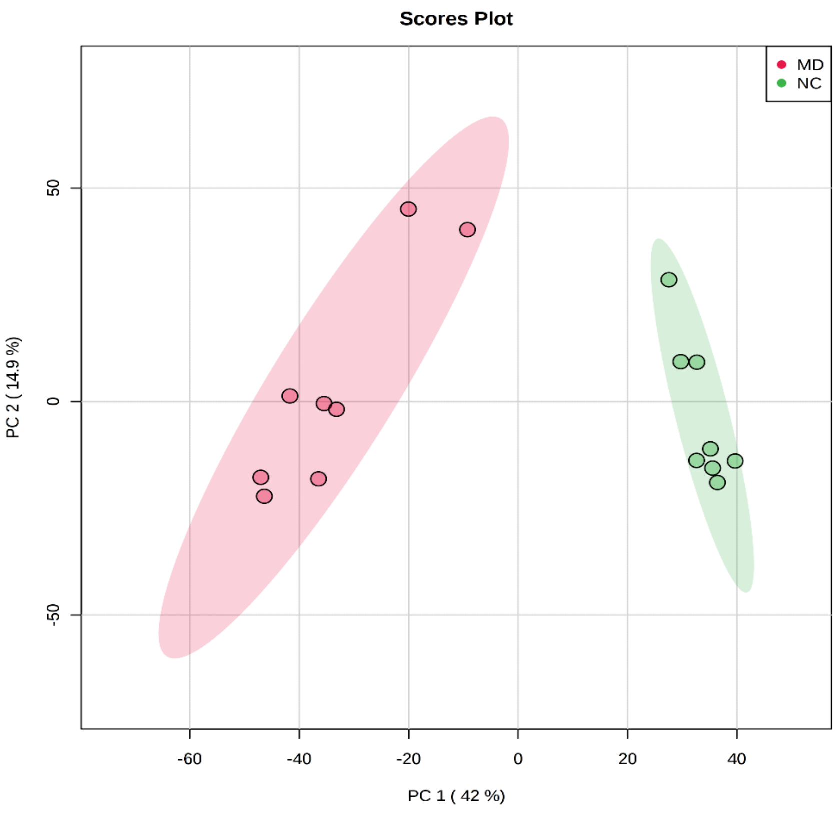

Figure S4. A diagram showing the L-Urobilin metabolism pathway.

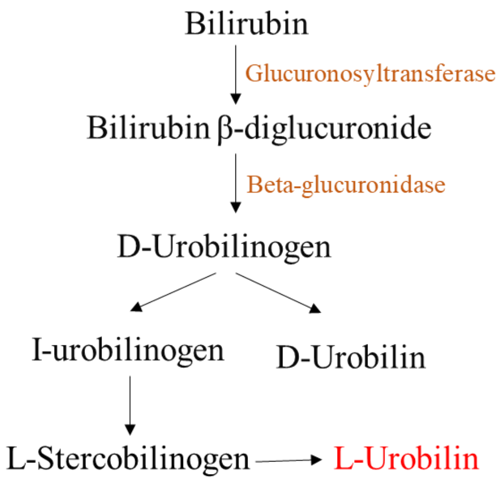

Figure S5. The microbial histidine kinase and histidine metabolism pathway significantly affected by neoagarotetraose supplementation NC: Normal controls; MD: DSS-induced colitis mice; NT: the DSS-induced colitis mice supplemented with a daily dose of 150 mg/kg bodyweight of neoagarotetraose for a total of 51 days.

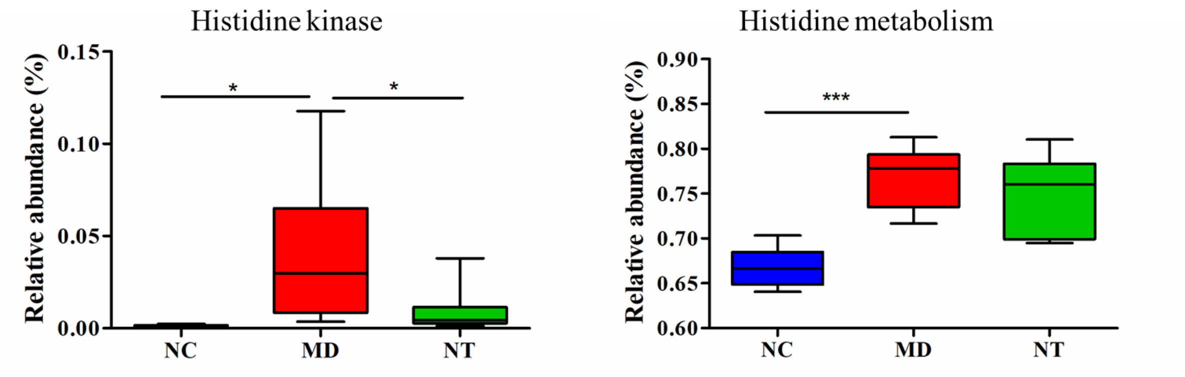

Table S1: Primers used in the study.

Gene symbol	Ref_gene_id	Forward primer	Reverse Primer
Il10	NM_010548.2	GGTTGCCAAGCCTTATCGGA	ACCTGCTCCACTGCCTTGCT
Il17a	NM_010552.3	ATCCCTCAAAGCTCAGCGTGTC	GGGTCTTCATTGCGGTGGAGAG
Il1b	NM_008361.4	CCAGCTTCAAATCTCACAGCAG	CTTCTTTGGGTATTGCTTGGGATC
Il4	NM_021283.2	ACAGGAGAAGGGACGCCAT	GAAGCCCTACAGACGAGCTCA
Il5	NM_010558.1	AGCACAGTGGTGAAAGAGACCTT	TCCAATGCATAGCTGGTGATTT
Il6	NM_031168.2	TCCAGTTGCCTTCTTGGGAC	GTACTCCAGAAGACCAGAGG
Nos2	NM_010927.4	CGAAACGCTTCACTTCCAA	TGAGCCTATATTGCTGTGGCT
Tlr4	NM_021297.3	GCCTTTCAGGGAATTAAGCTCC	AGATCAACCGATGGACGTGTAA
Tlr9	NM_031178.2	ACTCCGACTTCGTCCACCT	GGCTCAATGGTCATGTGGCA
Tnf	NM_013693.3	CCCTCACACTCAGATCATCTTCT	GCTACGACGTGGGCTACAG

Table S2. Select metabolites related to histidine metabolism were dysregulated by DSS. NC: normal controls; MD: colitis induced by DSS; NT: DSS induced colitis mice supplemented with NT.

Histidine Metabolism	MD/NC		NT/MD	
Metabolites	Fold change	*p*_value	Fold change	*p*_value
histamine	24.67	0.0000	0.82	0.5295
1-methylhistamine	66.05	0.0000	0.81	0.6801
hydantoin-5-propionate	22.91	0.0000	1.25	0.3606
3-methylhistidine	57.04	0.0000	0.77	0.5623
1-methyl-5-imidazoleacetate	0.13	0.0000	0.50	0.1617
formiminoglutamate	3.44	0.0001	0.77	0.3395
N-acetyl-3-methylhistidine	14.45	0.0003	1.38	0.2903
N-acetyl-1-methylhistidine	67.52	0.0009	1.01	0.5444
1-ribosyl-imidazoleacetate	62.47	0.0009	0.70	0.8839
histidine	2.81	0.0012	0.88	0.9238
imidazole lactate	13.79	0.0039	0.95	0.7844
trans-urocanate	0.54	0.0046	1.14	0.4428
imidazole propionate	3.87	0.0073	1.49	0.1978
N-acetylhistamine	4.72	0.0075	0.35	0.2632
1-methyl-4-imidazoleacetate	2.01	0.1187	0.89	0.9616
4-imidazoleacetate	1.16	0.2186	1.50	0.0605
cis-urocanate	0.79	0.1416	2.16	0.0074
N-acetylhistidine	1.48	0.5811	0.81	0.5049

Table S3. Select metabolites belonging to tocopherol metabolism were significantly affected by NT supplementation. NC: normal controls; MD: colitis induced by DSS; NT: DSS induced colitis mice supplemented with NT.

**Tocopherol Metabolism**	MD/NC		NT/MD	
Metabolites	Fold change	*p*_value	Fold change	*p*_value
delta-tocopherol	0.43	0.0021	1.45	0.0307
gamma-tocopherol/beta-tocopherol	0.13	0.0000	1.44	0.0111
alpha-tocopherol	0.52	0.0003	1.31	0.0209
alpha-tocopherol acetate	0.62	0.0048	2.13	0.1165
delta-CEHC	0.33	0.0226	1.40	0.2739
gamma-CEHC	0.45	0.0472	1.19	0.4157
gamma-CEHC sulfate	20.16	0.1709	0.97	0.7704
